# New formulation of the Gompertz equation to describe the kinetics of untreated tumors

**DOI:** 10.1371/journal.pone.0224978

**Published:** 2019-11-12

**Authors:** Antonio Rafael Selva Castañeda, Erick Ramírez Torres, Narciso Antonio Villar Goris, Maraelys Morales González, Juan Bory Reyes, Victoriano Gustavo Sierra González, María Schonbek, Juan Ignacio Montijano, Luis Enrique Bergues Cabrales

**Affiliations:** 1 Departamento de Matemática Aplicada, Instituto Universitario de Matemáticas y Aplicaciones, Universidad de Zaragoza, Zaragoza, Spain; 2 Departamento de Telecomunicaciones, Facultad de Ingeniería en Telecomunicaciones Informática y Biomédica, Universidad de Oriente, Santiago de Cuba, Cuba; 3 Departamento de Biomédica, Facultad de Ingeniería en Telecomunicaciones Informática y Biomédica, Universidad de Oriente, Santiago de Cuba, Cuba; 4 Universidad Autónoma de Santo Domingo, Santo Domingo, Dominican Republic; 5 Universidad Católica Tecnológica del CIBAO, Ucateci, La Vega, Dominican Republic; 6 Departamento de Ciencia e Innovación, Centro Nacional de Electromagnetismo Aplicado, Universidad de Oriente, Santiago de Cuba, Cuba; 7 Departamento de Farmacia, Facultad de Ciencias Naturales y Exactas, Universidad de Oriente, Santiago de Cuba, Cuba; 8 ESIME-Zacatenco, Instituto Politécnico Nacional, CD-MX, Mexico; 9 Grupo de las Industrias Biotecnológica y Farmacéuticas (BioCubaFarma), La Habana, Cuba; 10 Department of Mathematics, University of California Santa Cruz, Santa Cruz, CA, United States of America; Universita degli Studi di Genova, ITALY

## Abstract

**Background:**

Different equations have been used to describe and understand the growth kinetics of undisturbed malignant solid tumors. The aim of this paper is to propose a new formulation of the Gompertz equation in terms of different parameters of a malignant tumor: the intrinsic growth rate, the deceleration factor, the apoptosis rate, the number of cells corresponding to the tumor latency time, and the fractal dimensions of the tumor and its contour.

**Methods:**

Furthermore, different formulations of the Gompertz equation are used to fit experimental data of the Ehrlich and fibrosarcoma Sa-37 tumors that grow in male BALB/c/Cenp mice. The parameters of each equation are obtained from these fittings.

**Results:**

The new formulation of the Gompertz equation reveals that the initial number of cancerous cells in the conventional Gompertz equation is not a constant but a variable that depends nonlinearly on time and the tumor deceleration factor. In turn, this deceleration factor depends on the apoptosis rate of tumor cells and the fractal dimensions of the tumor and its irregular contour.

**Conclusions:**

It is concluded that this new formulation has two parameters that are directly estimated from the experiment, describes well the growth kinetics of unperturbed Ehrlich and fibrosarcoma Sa-37 tumors, and confirms the fractal origin of the Gompertz formulation and the fractal property of tumors.

## Introduction

One of the most interesting problems of current oncology is the understanding of the growth kinetics of a malignant tumor, named TGK (TGK), which follows a sigmoidal law. The TGK analysis is equally made by means of graphs of the number of cancer cells (n) versus time t, named n(t); tumor volume (V) versus t, named V(t); and/or the tumor mass (m) versus t, named m(t). This is due to the close relationship between these three physical quantities. Additionally, the sigmoidal form of TGK has been described by different equations, such as Gompertz, Logistics, Bertalanffy-Richards, Kolmogorov-Johnson-Mehl-Avrami modified, being the Gompertz equation (GE) the most used [1–3].

Izquierdo-Kulich et al. [4] report the fractal origin of GE (see appendix A). This fractal origin has also been reported in [5–8] but in terms only of the fractal dimension *D*_*f*_. Here, we have considered the one in [4] because it also takes into account the fractal structure of the boundary of the tumor.

In the different formulations of the GE [1–3] and in the experiment [9, 10] the starting point of TGK is considered when the initial number of tumor cells (n_0_) and the initial tumor volume (V_0_) satisfy the conditions n (t = 0) = n_0_ and V (t = 0) = V_0_, respectively. In preclinical studies, the researcher chooses n_0_/V_0_ depending on the purpose of the investigation. The time that elapses from the inoculation of the tumor cells in the host until the tumor reaches n_0_/V_0_ is named t_0_ [1, 3, 9]. Nevertheless, in clinics, n_0_/V_0_ corresponds to the tumor detected for the first time by the doctor by means of clinical and/or imaging methods. For this case, t_0_ is the time that elapses from the tumor formation in the organism (via chemical, biological and/or physical carcinogens) [10], until its detection for the first time. This supposes n_0_ ≥ n_med_, where n_med_ is the minimum number of quantifiable cancer cells contained in the smallest measurable tumor volume, named V_med_ (V_0_ ≥ V_med_). The post-inoculation time that elapses until the tumor reaches n_med_/V_med_ is named t_med_ (t_0_ ≥ t_med_) [3].

In [4], it is considered the Gompertz equation given in Eq (1) (named GE_1_)

$$n(t)=e^{\left(\frac{\alpha }{\beta }\right)\left(1-e^{-\beta t}\right)}.$$ (1)

According the considerations in the previous paragraph, GE_1_ has two limitations: 1) n_0_ = 1, which means that the tumor has only one cell when it reaches V_0_, in contradiction with the experiment [9, 10]. 2) The maximum capacity of the tumor (n_∞_) depends only on α and β and not on n_0_ (*n*(*t*) = *n*_∞_ = *e*^*α*/*β*^ when t → ∞). From the mathematical point of view, n_∞_ is the upper asymptote of TGK. Nevertheless, in the preclinical, the condition t → ∞ is the post-inoculation time that elapses until the tumor reaches a certain volume, for which animals are sacrificed for ethical reasons [1]. In clinics, this condition means the time that elapses from the tumor formation in the organism until the patient dies.

Each undisturbed solid tumor histological variety, that grows in a type of syngeneic host to it, has its own natural history (only sigmoidal law), which does not depend on the selection of n_0_/V_0_, as observed in [3, 10–12]. In the experiment, once the researcher fixes n_0_/V_0_, t_0_ can be estimated *a priori* when the tumor latency time is known, named t_obs_ (t_obs_ < t_0_), which is the post-inoculation time that elapses until that the tumor is observed for the first time. In this case, the tumor is observable and palpable but not measurable. However, its size, named V_obs_ (V(t = t_obs_) = V_obs_), is estimated following the methodology reported in [1, 3]. When the tumor reaches V_obs_, it contains a number of cells, named n_obs_ (n(t = t_obs_) = n_obs_).

The interest of including n_obs_/V_obs_ (n_obs_/V_obs_ < n_med_/V_med_ ≤ n_0_/V_0_) in GE is because an important part of vital cycle of a solid tumor occur before it is clinically detected (V_med_), as reported in [1, 3, 10]. Furthermore, a high cellular viability (≥ 95%) and a correct inoculation of the initial concentration of tumor cells (c_o_) are guaranteed, t_obs_ can be known *a priori* for a tumor histological variety that grows in a certain type of syngeneic host to it [3, 9–11].

As far as we reviewed, few experimental works report the analysis of TGK from V_obs_ [1, 3] and none of equations used to describe TGK includes n_obs_/V_obs_. In addition, in the literature a relationship of α and β in terms of D_f_, d_f_ and n_obs_/V_obs_ has not been reported in the literature. Therefore, the aim of this paper is to propose a new formulation of the GE that includes n_obs_/V_obs_, n_0_/V_0_, α, β, and to study the relation of these parameters with the fractal dimensions D_f_ and d_f_. The validity of this new mathematical formulation and the estimation of its parameters are determined from volumes of the Ehrlich and fibrosarcoma Sa-37 tumors that grow in BALB/c/Cenp mice, previously reported in [9]. Furthermore, the graphs of α versus d_f_ and β versus d_f_/D_f_ for different values of u_2_ (the constant of the velocity of apoptosis) and n_obs_ are shown.

## Methods

### Conventional Gompertz equation

Eq (2), named GE_2_, is the conventional GE and the most used when TGK starts at n_0_/V_0_, given by

$$n(t)=n_{0}\;e^{\left(\frac{\alpha }{\beta }\right)\left(1-e^{-\beta t}\right)}.$$ (2)

According to GE_2_, n_∞_ depends on n_0_, α and β (*n*(*t*) = *n*_∞_ = *n*_0_ *e*^*α*/*β*^ when t → ∞) and results from solving the ordinary differential Eq (3) with its initial condition, given by

$$\left\{ \begin{array}{l} \frac{dn}{dt}=\alpha n-\beta n\ln\frac{n}{n_{0}}=\alpha n\left(1-\frac{\beta }{\alpha }\ln\frac{n}{n_{0}}\right)\\ n(t=0)=n_{0} \end{array}\right..$$ (3)

GE_2_ suggests that n_0_ (constant in time) has to be included in Eq (A2). Tjørve and Tjørve [2] report that n_0_ acts as a parameter of shape (n_∞_ changes with n_0_) or location (n_∞_ remains constant).

### Inclusion of n_0_ in Eq (A2)

In this topic was followed the methodology exposed in [4] and the initial number of tumor cells at t = 0, named n_00_, was included in Eq (A2), resulting the following problem

$$\left\{ \begin{array}{l} \frac{d\ln(n)}{dt}=u_{2}(\theta -1)\ln\left(\frac{n}{n_{ss}}\right)\\ \ln{\left(n\right)}_{t=0}=\ln(n_{00})\;n(t=0)=n_{00} \end{array}\right..$$ (4)

The exact solution of Eq (3) was given by

$$n(t)={\left(n_{00}\right)}^{e^{-\beta t}}\;e^{\left(\frac{\alpha }{\beta }\right)\left(1-e^{-\beta t}\right)},$$ (5)

with

$$\left\{ \begin{array}{l} \alpha =u_{2}\left[\ln\frac{U_{1}}{u_{2}}\right]=u_{2}\ln\left(\frac{\frac{2}{3}d_{f}-1}{d_{f}-1}\right)\\ \beta =u_{2}(1-\theta )=u_{2}\left(1-\frac{d_{f}}{D_{f}}\right) \end{array}\right..$$ (6)

Two inconsistencies were found in [4]: 1) the coefficient 1.5 in the parameter α of Eq (A3) was not correct but 2/3, as in Eq (6). 2) Different types of experimental tumors with the same values of d_f_ and D_f_ had different values of α/β (we refer to the reader see Table 1 of [4]), in contrast to Eq (A3).

**Table 1:** Parameters of the models for the Ehrlich tumor.

Parameters	Different formulations of Gompertz equations			
	GE_1_	GE_2_	GE_5_	GE_8_
α (days^-1^)	0.160±0.005	0.466±0.012	0.285±0.004	0.719±0.067
β (days^-1^)	0.122±0.007	0.261±0.007	0.261±0.007	0.261±0.007
V_obs(α,β)_ (cm^3^)	-	-	-	0.190±0.063
u_2_ (days^-1^)	0.263±0.066	0.633±0.141	0.391±0.055	0.687±0.131
d_f_	0.720±0.061	0.768±0.056	0.764±0.032	0.611±0.052
D_f_	1.467±0.410	1.404±0.346	1.583±0.836	1.023±0.192
V_obs(u2,df,Df)_ (cm^3^)	-	-	-	0.190±0.041
α_c_ (days^-1^)	0.163±0.003	0.471±0.009	0.286±0.005	0.724±0.055
β_c_ (days^-1^)	0.134±0.104	0.287±0.005	0.275±0.009	0.261±0.007
SE	0.215±0.006	0.884±0.021	0.088±0.021	0.089±0.021
PRESS	1.313±0.154	0.015±0.012	0.015±0.012	0.016±0.012
MPRESS	1.128±0.144	0.015±0.012	0.015±0.012	0.016±0.012
*r*^2^	0.990±0.006	0.998±0.009	0.998±0.009	0.998±0.001
$r_{a}^{2}$	0.990±0.006	0.998±0.009	0.998±0.009	0.998±0.001
RMSE (cm^3^)	0.214±0.006	0.088±0.021	0.087±0.021	0.088±0.022
D_max_ (cm^3^)	0.501±0.013	0.194±0.050	0.194±0.050	0.195±0.050
e_α_	0.042±0.015	0.073±0.030	0.053±0.021	0.095±0.047
e_β_	0.040±0.018	0.046±0.019	0.048±0.022	0.047±0.020
e_Vobs(α,β)_	-	-	-	0.033±0.009
e_u2_	0.046±0.007	0.052±0.023	0.051±0.013	0.082±0.025
e_df_	0.071±0.011	0.072±0.019	0.070±0.021	0.073±0.020
e_Df_	0.325±0.075	0.415±0.068	0.761±0.108	0.054±0.014
e_Vobs(u2,df,Df)_	-	-	-	0.032±0.008

Means ± standard deviation of parameters of the Ehrlich tumor and criteria for model assessment obtained for different formulations of Gompertz equations.

Eq (5), named GE_5_, agrees with GE_2_ when $n_{0}={\left(n_{00}\right)}^{e^{-\beta t}}$. In addition, the parameters n_00_ and n_0_ coincided exactly at t = 0. The constant parameter n_00_ (n_00_ ≥ n_med_) constituted the starting point of TGK for GE_5_ and reached for t = t_0_. Therefore, it was convenient to differentiate n_0_ and n_00_ to compare GE_2_ and GE_5_ in order to avoid confusion in the interpretation of these two parameters. GE_5_ revealed that n_∞_ depends only on α and β and not on n_00_ (*n*(*t*) = *n*_∞_ = *e*^*α*/*β*^ for t → ∞).

### Inclusion of n_obs_ in GE

Eq (3) was rewritten as

$$\left\{ \begin{array}{l} \frac{dn}{dt}=\alpha n\left(1-\frac{\beta }{\alpha }\ln\frac{n}{n_{obs}}\right)\\ n(t=0)=n_{000} \end{array}\right.,$$ (7)

where n_000_ was the number of tumor cells that the researcher selected at t = t_0_. The analytical solution of Eq (7) was given by

$$n(t)=\left[n_{obs}{\left(\frac{n_{000}}{n_{obs}}\right)}^{e^{-\beta t}}\right]e^{\left(\frac{\alpha }{\beta }\right)\left(1-e^{-\beta t}\right)}.$$ (8)

Eq (8), named GE_8_, agreed with GE_5_ at t = 0 (for all n_obs_) and when n_obs_ = 1 (for all t). The GE_8_ coincided with the GE_2_ at t = 0 (for all n_obs_) and when $n_{0}=n_{obs}{\left(n_{000}/n_{obs}\right)}^{e^{-\beta t}}$. The parameter n_obs_ (n_obs_ < n_med_ ≤ n_000_) was the starting point of TGK. In general, n_000_ did not coincide with n_0_ (GE_2_) or n_00_ (GE_5_). Therefore, it was convenient to differentiate the parameters n_0_, n_00_ and n_000_. In addition, the GE_8_ evidenced that n_∞_ depends on n_obs_, α and β, but not on n_000_ (*n*(*t*) = *n*_∞_ = *n*_*obs*_ *e*^*α*/*β*^ for t → ∞). The parameters α and β in terms of u_2_, U_1_, θ, d_f_, D_f_ and n_obs_ were given by

$$\left\{ \begin{array}{l} \alpha =u_{2}\left[\ln\frac{U_{1}}{u_{2}}\right]-\beta \ln(n_{obs})=u_{2}\ln\left(\frac{\frac{2}{3}d_{f}-1}{d_{f}-1}\right)-\beta \ln(n_{obs})\\ \beta =u_{2}(1-\theta )=u_{2}\left(1-\frac{d_{f}}{D_{f}}\right) \end{array}\right..$$ (9)

Eq (9) resulted from assuming that the value of n in the steady state was *n*_*ss*_ = *n*_*obs*_ *e*^*α*/*β*^ = (*u*_2_/*U*_1_)^1/(*θ*−1)^ and Eqs (7) and (8) were taken into account.

### Simulations

#### Simulation of Eq (9)

Eq (9) coincided with Eq (6) for n_obs_ = 1. The simulation of α (in days^-1^) versus d_f_ was shown for D_f_ = 5 and four values for u_2_ (1, 10, 50 and 100 days^-1^) and n_obs_ (1, 5, 10 and 20 cells). For this, values of d_f_ were varied from 0 to 5 with a step of 0.5, taking into account that d_f_ < D_f_. The simulation of β (in days^-1^) against d_f_/D_f_ was shown for four values of u_2_ (1, 10, 50 and 100 days^-1^) and the values of d_f_/D_f_ were ranged from 0 to 5 with a step of 0.5.

#### Simulations of GE_2_, GE_5_ and GE_8_

GE_5_ was used as reference because GE_5_ and GE_8_ were reported for the first time in the literature. The simulations of GE_2_, GE_5_ and GE_8_ were shown in a graph of n(t). Simulation of GE_2_ was made for different values of n_0_ (1x10^3^, 1x10^4^, 1x10^5^ and 1x10^6^ cells). Additionally, GE_8_ was simulated for three different situations: 1) n_obs_ = 1 cell (GE_8_ and GE_5_ coincided) and different values of n_00_ (5, 10, 15, 20 and 25 cells); 2) n_obs_ = 1x10^4^ cells and different values of n_000_ (1x10^4^, 5x10^4^, 1x10^5^ and 2x10^5^ cells); and 3) n_000_ = 1x10^5^ cells and different values of n_obs_ (5x10^3^, 1x10^4^, 5x10^4^ and 1x10^5^ cells). In all these simulations, α = 1.0 days^-1^ and β = 0.3 days^-1^.

### Experimental groups

In this study, V(t) was used by three reasons: 1) V(t) is related to n(t) and can be used interchangeably; 2) V(t) is less cumbersome to estimate than n(t) and it is frequently used in preclinical [9–11] and clinical [10] studies; and 3) the graphs of V(t) and n(t) shown sigmoidal shapes. Consequently, n(t) in GE_1_, GE_2_, GE_5_ and GE_8_ was replaced by V(t); n_0_ in GE_2_ by V_0_; n_00_ in GE_5_ by V_00_; n_000_ and n_obs_ in GE_8_ by V_000_ and V_obs_, respectively. In addition, n_med_ was replaced by V_med_ and n_∞_ by V_∞_. The parameter V_∞_ was the tumor volume when t → ∞.

Two experimental groups were formed, each consisting of 10 male BALB/c/Cenp mice. The first group corresponded to the Ehrlich tumor, denominated G1, while the second group to the fibrosarcoma Sa-37 tumor, denominated G2. Experimental data of V(t) for Ehrlich and fibrosarcoma Sa-37 tumors were reported in [9], corresponding to their control groups.

### Interpolation of experimental data

The Hermite interpolation method [13] was used to interpolate volume data of each individual tumor, in G1 and G2.

### Estimation of values of α, β, d_f_, D_f_ and u_2_ from experimental data

Values of α and β (GE_1_, GE_2_, GE_5_ and GE_8_) and V_obs_ (GE_8_) were obtained from the individual fitting of each tumor volume (Ehrlich and fibrosarcoma Sa-37). The value of V_obs_ estimated directly with GE_8_ was named V_obs(α,β)_. The value V_0_ = V_00_ = V_000_ = 0.5 cm^3^ was the tumor volume chosen to describe TGK. This volume value was reached 15 days after 2x10^6^ cells for the Ehrlich tumor and 5x10^5^ cells for the fibrosarcoma tumor Sa-37 were inoculated in the BALB/c/Cenp mouse (see details in [9]).

Three equations in terms of d_f_, D_f_ and u_2_ resulted when Eq (6) was substituted in GE_1_, GE_2_ and GE_5_. The values of these three parameters were determined when each of these equations was used to fit experimental data. Besides, Eq (12) was substituted in GE_8_ and resulted an equation in terms of d_f_, D_f_, u_2_ and V_obs_, from which their values were estimated from fitting experimental data. Once known the values of d_f_, D_f_, u_2_ and V_obs_, they were substituted in their respective Eqs (6) and (9) to calculate their corresponding values of α and β. Values of α, β and V_obs_ obtained by this way were denominated α_c_, β_c_ and V_obs(u2,df,Df)_, respectively, to distinguish these values from those that were directly obtained from fitting of the experimental data with GE_1_, GE_2_, GE_5_ and GE_8_.

The estimation errors for α, β, d_f_, D_f_, u_2_, V_obs_ and V_obs(u2,df,Df)_ were denominated e_α_, e_β_, e_df_, e_Df_, e_u2_, e_Vobs_ and e_Vobs(u2,df,Df)_, respectively. The estimation error for each parameter was reported for each individual tumor of Ehrlich and fibrosarcoma Sa-37.

The difference between α and α_c_, named Δα (Δα = α—α_c_), was calculated for each equation (GE_1_, GE_2_, GE_5_ and GE_8_) and experimental group (G1 and G2). In addition, it were computed differences between β and β_c_, denominated Δβ (Δβ = β—β_c_), and V_obs(_u_2_,d_f_,D_f)_ and V_obs(α,β)_, denominated ΔV_obs_ (ΔV_obs_ = V_obs(α,β)_—V_obs(_u_2_,d_f_,D_f)_).

### Criteria for model assessment

Five quality-of-fit criteria were used for fitting of experimental data with GE_1_, GE_2_, GE_5_ and GE_8_: the sum of squares of errors, SSE (Eq (10)); standard error of the estimate, SE (Eq (11)); adjusted goodness-of-fit coefficient of multiple determination, $r_{a}^{2}$ (Eq (12)), that depended on goodness-of-fit coefficient *r*^2^ (Eq (14)); predicted residual error sum of squares, PRESS (Eq (14)); and multiple predicted residual sum error of squares, MPRESS (Eq (15)) [1, 3, 14], given by

$$SSE=\sum_{j=1}^{n_{1}}{\left({\hat{V}}_{j}^{*}-V_{j}^{*}\right)}^{2},$$ (10)

$$SE=\sqrt{\frac{\sum_{j=1}^{n_{1}}{\left({\hat{V}}_{j}^{*}-V_{j}^{*}\right)}^{2}}{n_{1}-k}},$$ (11)

$$r_{a}^{2}=1-\frac{n_{1}-1}{n_{1}-k}\left(1-r^{2}\right)=\frac{\left(n_{1}-1\right)r^{2}-k+1}{n_{1}-k},$$ (12)

$$1-r^{2}=\frac{\sum_{j=1}^{n_{1}}{\left({\hat{V}}_{j}^{*}-V_{j}^{*}\right)}^{2}}{\sum_{j=1}^{n_{1}}{\left(V_{j}^{*}\right)}^{2}-\frac{1}{n_{1}}{\left(\sum_{j=1}^{n_{1}}V_{j}^{*}\right)}^{2}},$$ (13)

$$PRESS=\frac{\sum_{j=1}^{n_{1}-1}{\left[{\left({\hat{V}}_{j}^{*}\right)}^{´}-V_{j}^{*}\right]}^{2}}{n_{1}-k},$$ (14)

$$MPRESS(m)=\frac{\sum_{j=m+1}^{n_{1}}{\left[{\left({\hat{V}}_{j}^{*}\right)}^{´}-V_{j}^{*}\right]}^{2}}{n_{1}-m},$$ (15)

where $V_{j}^{*}$ was the *j*-th measured tumor volume at discrete time *t*_*j*_, j = 1, 2, …, n_1_; ${\hat{V}}_{j}^{*}$ was the *j*-th estimated tumor volume by GE_1_, GE_2_, GE_5_ and GE_8_; *n*_1_ the number of experimental points (n_1_ = 10) and *k* the number of parameters (k = 2 for GE_1_, GE_2_ and GE_5_, and k = 3 for GE_8_). The fitting was considered to be satisfactory when $r_{a}^{2}>0.98$. Higher $r_{a}^{2}$ meant a better fit. ${\left(V_{j}^{*}\right)}^{´}$ was the estimated value of $V_{j}^{*}$ when GE_1_/GE_2_/GE_5_/GE_8_ was obtained without the *j*-th observation. MPRESS removed the last *n*_1_−*m* measurements. Each equation (GE_1_, GE_2_, GE_5_ and GE_8_) was fitted to the first m measured experimental points (m = 3, 4 or 5) and then from calculated model parameters the error between tumor volume estimated and measured values in the remaining *n*_1_−*m* points was calculated. Least Sum of Squares of Errors was obtained when SSE was minimized in the Marquardt-Levenberg optimization algorithm.

The Root Means Square Error, RMSE (Eq (16)) and the maximum distance, D_max_ (Eq (17)) were also calculated following the methodology suggested in [1, 3, 14], given by

$$RMSE=\sqrt{\sum_{i=1}^{M}\frac{{\left(F_{i}-G_{i}\right)}^{2}}{M}},$$ (16)

$$D_{m\overset{´}{a}x}=m\overset{´}{a}x|F_{i}-G_{i}|,$$ (17)

where M was the number of interpolated data of tumor kinetics (graph of V(t)). F_i_ was the *i-th* tumor volume of the experimental data, which was chosen as reference. G_i_ was the *i-th* tumor volume calculated with GE_1_, GE_2_, GE_5_ and GE_8_.

Each fit with the GE_1_/GE_2_/GE_5_/GE_8_ was performed for each animal growth curve. A computer program was implemented in the Matlab^®^ software (version R2012b 64-bit, Institute for Research in Mathematics and Applications, University of Zaragoza, Spain) to calculate the tumor volume. In addition, the mean ± standard error of each parameter of the equation (α, β, V_obs(α,β)_, u_2_, d_f_, D_f_, V_obs(u2,df,Df)_, α_c_, β_c_), fit criterion (SE, PRESS, MPRESS, $r_{a}^{2}$, RMSE and D_max_) and estimation error (e_α_, e_β_, e_df_, e_Df_, e_u2_, e_Vobs_ and e_Vobs(u2,df,Df)_) were calculated from their individual values, in each experimental group, following the methodology reported in [1, 3]. These calculations were performed on a PC with an Intel(R) core processor (TM) i7-3770 at 3.40 GHz with a Windows 10 operating system. All calculations took approximately 10 min, for each equation.

## Results

### Simulation of Eq (6)

**Fig 1:**
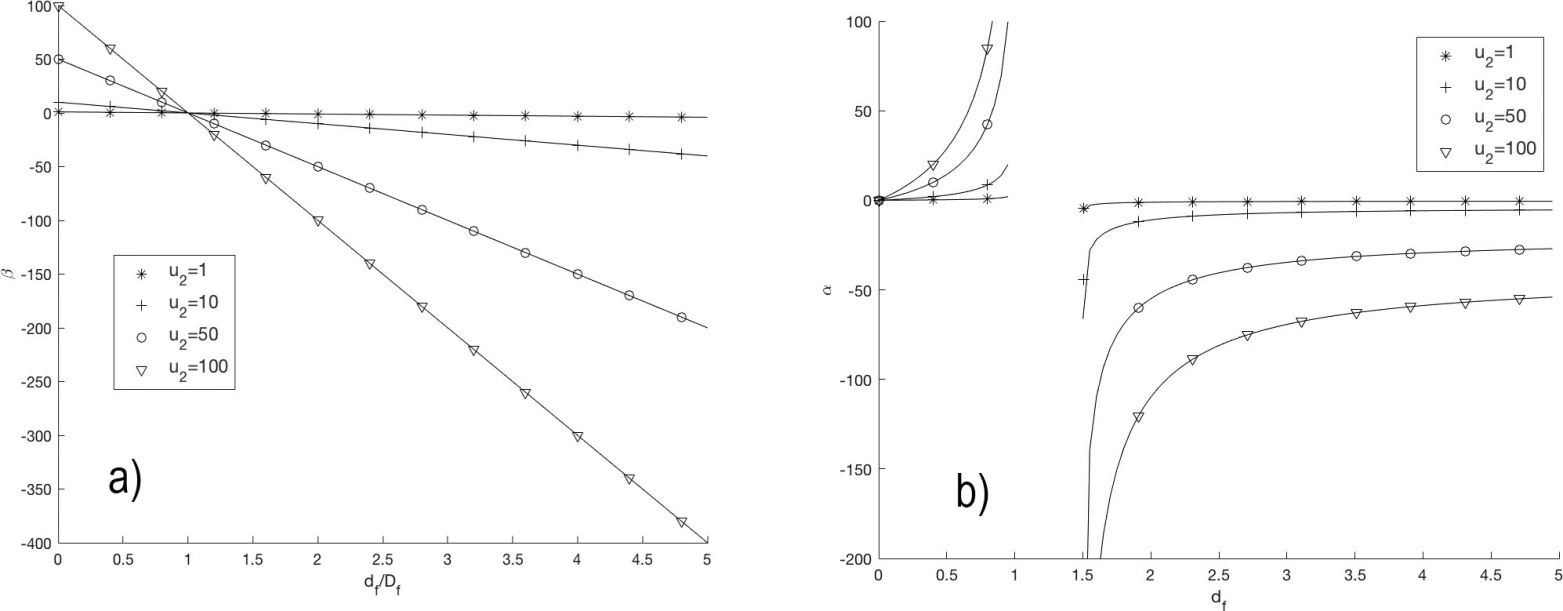
Simulation of Eq (6). For different values of u_2_ (1, 10, 50 and 100 days^-1^) it is plotted (*A*) Graph of α (in days^-1^) versus d_f_ and (*B*) Graph of β (in days^-1^) versus d_f_/D_f_.

Fig 1 showed the simulations of β versus d_f_/D_f_ (Fig 1*A*) and α versus d_f_ (Fig 1*B*) for different values of u_2_. The positive values of α (in the interval 0 ≤ d_f_ < 1) and β (in the interval d_f_/D_f_ < 1) increased non-linearly with the increase of d_f_ and decreased linearly with the increase of d_f_/D_f_, respectively. The negative values of α increased non-linearly with the increase in d_f_ (d_f_ > 1.5). The negative values of β decreased linearly with the increase of d_f_/D_f_ (d_f_/D_f_ > 1). These behaviors were noticeable for the greater value of u_2_. Additionally, the parameter α had a discontinuity in the interval 1 < d_f_ < 1.5 and β = 0 when d_f_/D_f_ = 1 for all values of u_2_.

### Simulation of Eq (9)

**Fig 2:**
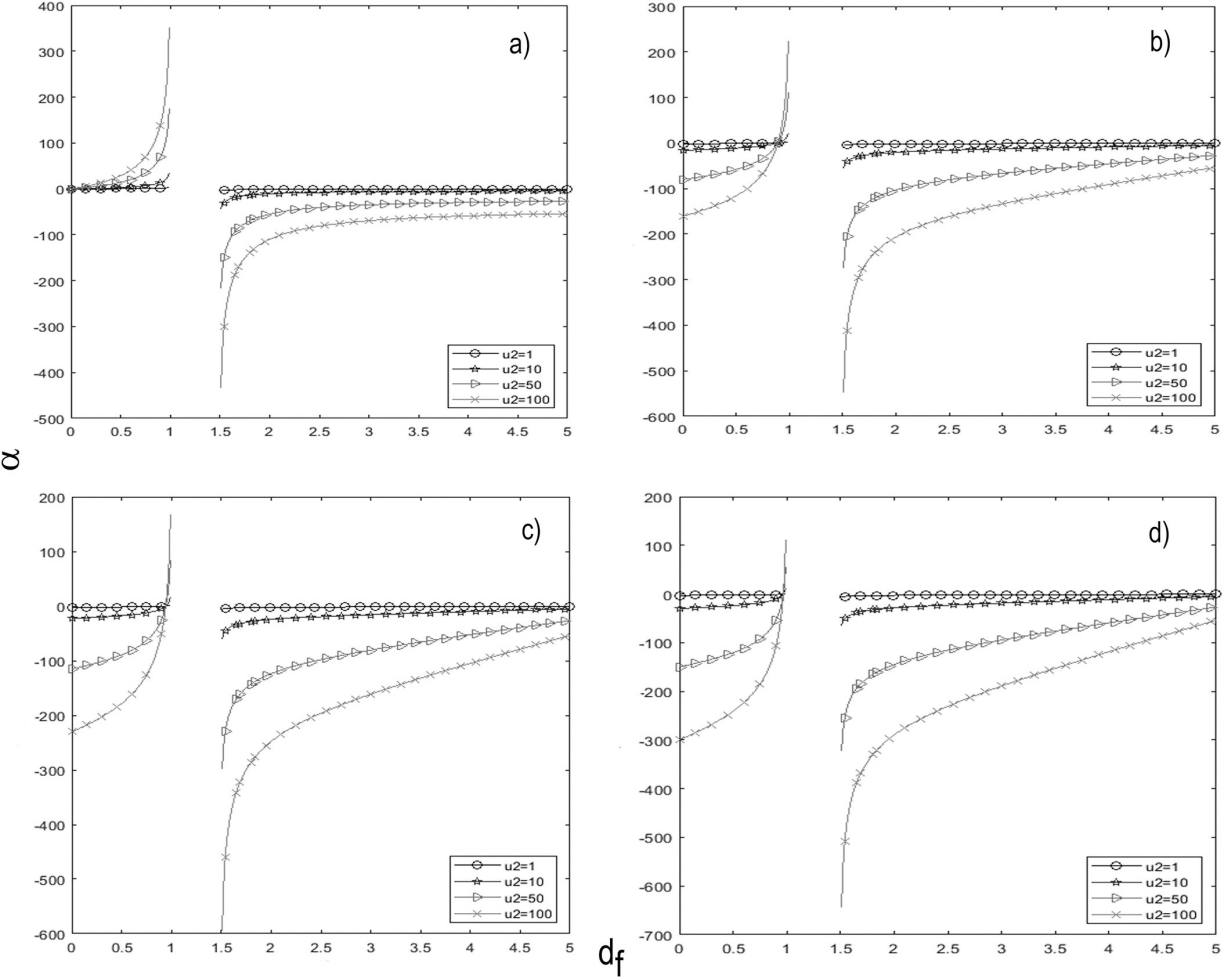
Simulation of Eq (9). For different values of u_2_ (1, 10, 50 and 100 days^-1^) it is plotted the graph of α (in days^-1^) versus d_f_ for (*A*) n_obs_ = 1 cell. (*B*) n_obs_ = 5 cells. (*C*) n_obs_ = 10 cells. (*D*) n_obs_ = 20 cells.

Results of the simulation of β versus d_f_/D_f_ in Eq (11) coincided with that shown in Fig 1*A* (see Eqs (6) and (9)). The simulation of α versus d_f_ for n_obs_ = 1 (Fig 2*A*) reproduced the same result as in Fig 1*B*. However, values of α were more negative, in the interval 0 ≤ d_f_ < 1, when n_obs_ increased, being noticeable for the higher value of u_2_ (Fig 2*B*, 2*C* and 2*D*). In Fig 2*A*, 2*B*, 2*C* and 2*D*, as in Fig 1*B*, it was observed a discontinuity of α in the interval 1 < d_f_ < 1.5.

### Simulations of GE_2_, GE_5_ and GE_8_

**Fig 3:**
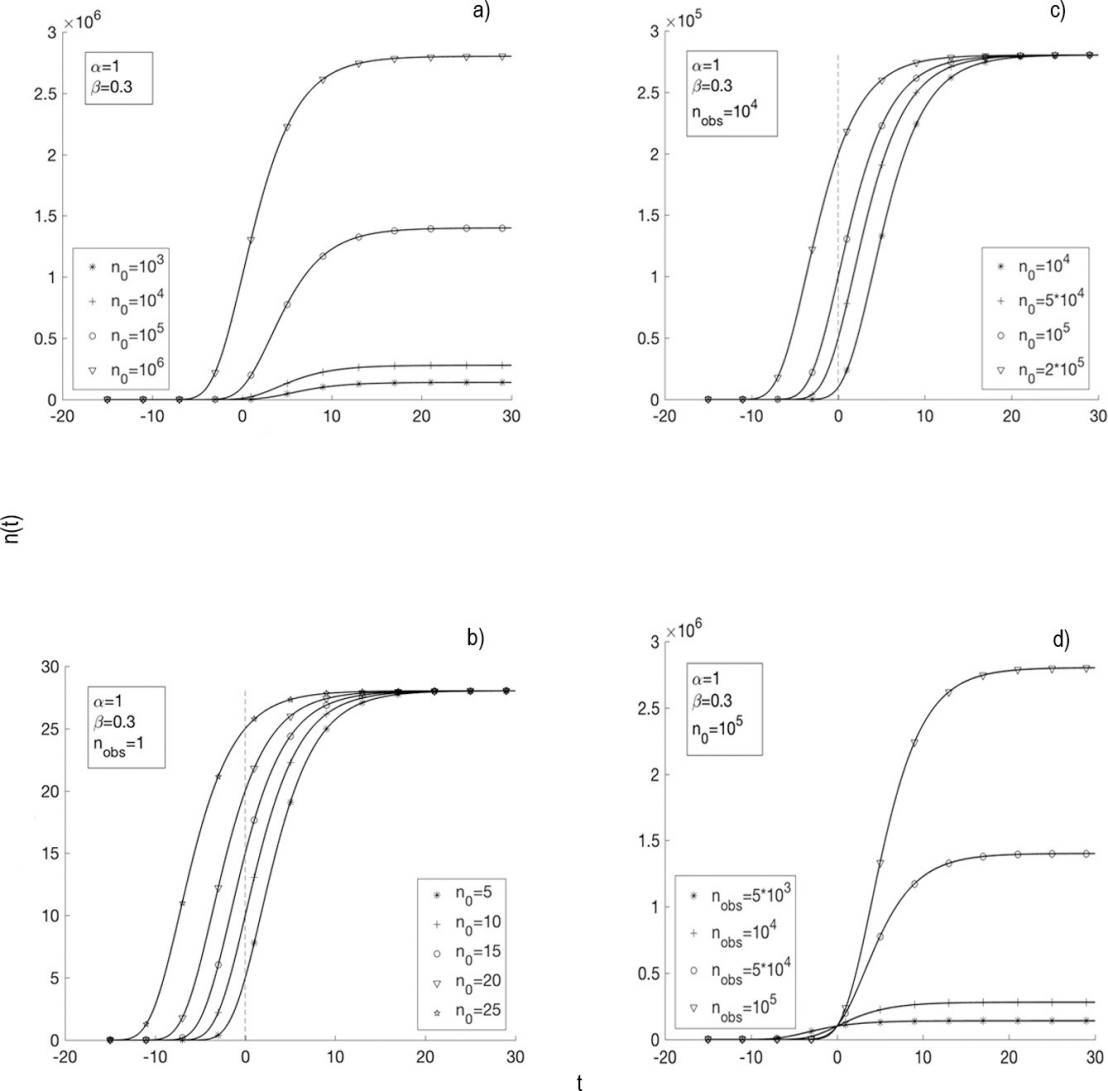
Evolution of the number of cells with time. Simulation of the number of cells at time t, in days, (n (t)) for α = 1.0 days^-1^ and β = 0.3 days^-1^. (*A*) Simulation of GE_2_ for different values of n_0_ (1x10^3^, 1x10^4^, 1x10^5^ and 1x10^6^ cells). (*B*) Simulation of GE_8_ for n_obs_ = 1 cell (coincides with GE_5_) and different values of n_00_ = n_0_ (5, 10, 15, 20 and 25 cells). (*C*) Simulation of GE_8_ for n_obs_ = 1x10^4^ cells and different values of n_000_ = n_0_ (1x10^4^, 5x10^4^, 1x10^5^ and 2x10^5^ cells). (*D*) Simulation of GE_8_ for n_000_ = n_0_ = 1x10^5^ cells and different values of n_obs_ (5x10^3^, 1x10^4^, 5x10^4^ and 1x10^5^ cells).

Fig 3 showed the behavior of n(t) when GE_2_ (Fig 3*A*, GE_5_ (Fig 3*B*) and GE_8_ (Fig 3*C* and 3*D*) were used. Fig 3*A* revealed that the highest value of n_∞_ and the fastest TGK occurred for the highest values of n_0_ and α. Fig 3*B* showed that TGK was faster with the increase of n_00_ and all TGK tended to the same value of n_∞_ for all value of n_00_, keeping constant values of α and β. In this case, TGK was faster when the value of n_00_ increased with respect to n_obs_ (Fig 3*B*), being noticeable when n_obs_ increased with respect to 1 (Fig 3*C*). It is important to note that n_0_ = n_00_ (Fig 3*B*) and n_0_ = n_000_ (Fig 3*C* and 3*D*).

The results of Fig 3*D* showed that TGK grows slower (when n < n_000_) and then faster (when n > n_000_) for the greater value of n_obs_; all TGK were cut at t = 0 (same value of n_000_), for all value of n_obs_; and the value of n_∞_ depended on n_obs_ and not n_000_ for each TGK. The results shown in Fig 3 were noticeable when the value of α increased with respect to that of β (results not shown).

### Fitting of experimental data with GE_1_, GE_2_, GE_5_ and GE_8_ and estimation of values of α, β, d_f_, D_f_ and u_2_

The mean ± standard deviation of each parameter of the equation, fit criterion and estimation error were shown in Tables 1 and 2 of each equation (GE_1_, GE_2_, GE_5_ and GE_8_) used to fit experimental data of the Ehrlich and fibrosarcoma Sa-37 tumors, respectively. Tables 1 and 2 shown for these two tumor histological varieties: 0 < d_f_ < 1; 1 < D_f_ < 2; 0 < u_2_ < 1; the highest values of α, u_2_ and the lowest values of d_f_ and D_f_ for GE_8_; the lowest SE values for GE_5_ and GE_8_; the lowest values of PRESS, MPRESS, RMSE and D_max_; the highest values of *r*^2^ and $r_{a}^{2}$ for GE_2_, GE_5_ and GE_8_; and values of the parameter α differed when GE_1_, GE_2_, GE_5_ and GE_8_ were used. Nevertheless, the parameter β was the same when GE_2_, GE_5_ and GE_8_ were used, but not for GE_1_.

**Table 2:** Parameters of the models for the fibrosarcoma Sa-37 tumor.

Parameters	Different formulations of Gompertz equations			
	GE_1_	GE_2_	GE_5_	GE_8_
α (days^-1^)	0.188±0.016	0.491±0.034	0.316±0.018	0.833±0.132
β (days^-1^)	0.127±0.017	0.252±0.018	0.252±0.018	0.252±0.018
V_obs(α,β)_ (cm^3^)	-	-	-	0.148±0.088
u_2_ (days^-1^)	0.274±0.093	0.530±0.152	0.471±0.132	0.576±0.070
d_f_	0.759±0.074	0.822±0.070	0.746±0.058	0.688±0.042
D_f_	1.704±0.672	1.810±0.612	1.837±0.613	1.256±0.191
V_obs(_u_2,_ d_f,_D_f)_ (cm^3^)	-	-	-	0.142±0.029
α_c_ (days^-1^)	0.197±0.020	0.494±0.029	0.322±0.011	0.814±0.082
β_c_ (days^-1^)	0.152±0.018	0.290±0.020	0.280±0.017	0.252±0.018
SE	0.162±0.008	0.082±0.038	0.083±0.038	0.083±0.038
PRESS	0.761±0.227	0.063±0.059	0.063±0.059	0.064±0.060
MPRESS	0.623±0.203	0.063±0.059	0.064±0.059	0.064±0.060
*r*^2^	0.995±0.004	0.998±0.001	0.998±0.001	0.999±0.001
$r_{a}^{2}$	0.996±0.004	0.998±0.001	0.998±0.001	0.999±0.001
RMSE (cm^3^)	0.161±0.008	0.082±0.038	0.082±0.038	0.082±0.038
D_max_ (cm^3^)	0.499±0.013	0.206±0.109	0.206±0.100	0.207±0.110
e_α_	0.025±0.011	0.046±0.022	0.061±0.012	0.079±0.035
e_β_	0.034±0.009	0.053±0.013	0.057±0.029	0.055±0.023
e_Vobs(α,β)_	-	-	-	0.027±0.007
e_u2_	0.031±0.003	0.035±0.013	0.039±0.010	0.061±0.015
e_df_	0.065±0.012	0.069±0.014	0.067±0.016	0.071±0.025
e_Df_	0.235±0.086	0.336±0.045	0.679±0.119	0.125±0.031
e_Vobs(u2,df,Df)_	-	-	-	0.041±0.017

Means ± standard deviation of parameters of the fibrosarcoma Sa-37 tumor and criteria for model assessment obtained for different formulations of Gompertz equations.

For the Ehrlich tumor, Δα = 0.003, 0.005, 0.001 and 0.005 days^-1^ for GE_1_, GE_2_, GE_5_ and GE_8_, respectively. The variable Δβ = 0.012, 0.026, 0.014 and 0.000 days^-1^ for these respective equations and ΔV_obs_ = 0.007 cm^3^. For the tumor fibrosarcoma Sa-37, Δα = 0.009, 0.003, 0.006 and 0.019 days^-1^ for GE_1_, GE_2_, GE_5_ and GE_8_, respectively. The variable Δβ = 0.025, 0.038, 0.028 and 0.000 days^-1^ for these respective equations and ΔV_obs_ = 0.006 cm^3^.

## Discussion

This study shows that GE_2_, GE_5_ and GE_8_ can be used interchangeably to describe experimental data of Ehrlich and fibrosarcoma Sa-37 tumors, taking into account their higher values of *r*^2^ and $r_{a}^{2}$, and lower values of each parameter of the equation, fit criterion, estimation error, Δα, Δβ and ΔV_obs_ (ΔV_obs_ is only calculated for GE_8_).

The theoretical and experimental results of this work confirm different findings reported previously in the literature, such as: 1) the fractal origin of GE_1_, GE_2_, GE_5_ and GE_8_, as reported in [4, 15]; 2) the fractal property of tumors once reached n_med_/V_med_, a matter that agrees with [16, 17]; 3) the role of the fractal dimension for the understanding of TGK, as suggested by Sokolov [18] and Breki et al. [19]; and 4) 1 < D_f_ < 2, in agreement with [4, 20, 21] and the preferential growth along the largest diameter of the tumor, despite its ellipsoidal geometry [1, 3, 9, 11]. This fourth finding is in contradiction with 2 < D_f_ < 3 reported by Breki et al. [19] in patients with metastatic melanoma; 5) The condition 0 < u_2_ < 1 for both types of tumors is consistent with the Steel equation [12]. If u_2_ = 0, then the tumor growth fraction must be high so that its mean doubling time (TD) is short, in contrast to [10, 12]. If u_2_ = 1 day^-1^ (all cancer cells are in apoptosis), TD → ∞ and α = 0 (tumor self-destruction), in contrast to the failure of the apoptosis mechanism in malignant tumors (because of the gene p-53 is repressed) and the existence of other cell loss mechanisms (metastasis, necrosis and exfoliation) [10, 11, 22]. The increase in u_2_ brings about a decrease in TD and therefore a higher value of α (Figs 1*B*, 2*A*, 2*B*, 2*C* and 2*D*).

Other novel findings have been revealed in this investigation that may be of interest for understanding of TGK, such as: 1) TGK sigmoidal form and n_∞_/V_∞_ do not depend on n_0_ and if on α, β and n_obs_/V_obs_, when a given tumor histological variety grows in a certain type of syngeneic host to it. In this way, the action form of parameter n_0_/V_0_ (form or location) is eliminated in GE_2_, as reported in [2]. 2) The GE_8_ states that n_0_ in the GE_2_ is not a constant parameter but depends non-linearly with n_obs_/V_obs_, n_000_/n_obs_ (V_000_/V_obs_), β and t. 3) The growth of a malignant tumor occurs for 0 < d_f_ < 1 and not when d_f_ = 0 (α = 0: the tumor does not form), 1 < d_f_ < 1.5 (discontinuity of α due to forbidden conformations or very unlikely tumor) and d_f_ > 1.5 (α < 0: the tumor self-destructs), in contrast to the values of d_f_ (1 < d_f_ < 2) reported in [4, 14, 23]. The forbidden conformations of the tumor can be explained by its stereochemistry due to the steric collides between all its elements and the tumor-host interaction. 4) The increase of α with the increase of d_f_, at 0 < d_f_ < 1, confirms that the growth efficiency of a malignant tumor increases with its d_f_, in agreement with [17, 24]. 5) Eq (11) states that this increase of α with d_f_ occurs if n_obs_ satisfies strictly the condition $n_{obs}<{\left[\left(2/3d_{f}-1\right)/\left(d_{f}-1\right)\right]}^{u_{2}/\beta }$; otherwise, α < 0 for all β positive (Fig 2B, 2*C* and 2*D*). The case α < 0 means that the tumor self-destructs, in contrast to the experiment.

The established condition for n_obs_ suggests that: 1) n_obs_/V_obs_ depends on d_f_ and the ratio u_2_/β; 2) the fractal property of a malignant tumor also happens before or long before its detection (n_med_/V_med_), as reported in [1, 25]; 3) the ratio u_2_/β may be an indirect indicator of the apoptosis-angiogenesis relationship reported in [26, 27]; 4) endogenous anti-angiogenic factors or inhibitors of angiogenesis (endostatin, angiostatin, among others) are present in the tumor before or long before reaching n_med_/V_med_; 5) the term *e*^−*βt*^ (see GE_8_ and the established condition for n_obs_) and the decrease of the parameter β with the increase of d_f_/D_f_ corroborate the essential role of angiogenesis process and the displacement of the balance between endogenous anti-angiogenic factors and endogenous pro-angiogenic factors towards these latter, when the tumor volume grows at time t, consistent with [10, 17, 22, 28, 29].

From the mathematical point of view, the condition 0 < d_f_ < 1 may suggest that the contours of Ehrlich and fibrosarcoma Sa-37 malignant tumors have zero area and/or they are totally disconnected. The first assumption confirms that these two types of tumors can be delimited from their surrounding healthy tissue, as in [9, 11]. The second hypothesis is based on proposition 2.5 [30]: “A set $F\subset {ℜ}^{n}$ with dim_*H*_ *F*<1 is totally disconnected”. In this proposition, F is any set and dim_H_ is the fractal dimension Hausdorff. It is important to note that, although the tumor boundary is wide, d_f_ < 1 if its only fractality is given by a totally disconnected line contained in that wide band.

From the biophysical point of view, the tumor contour totally disconnected can indicate the existence in it of pores/channels formed randomly of different sizes and shapes, changing in the time. This porous contour of a tumor may be related to the angiogenesis process (neo-formation of blood vessels), the formation of spicules by fragmentation of the contour into simple forms of molds (for example, triangles), roundness, irregular edge, anisotropy, roughness and compactness, findings reported in [1, 3, 10, 22, 31–34]. We believe that the tumor angiogenesis process can be regulated by the amount of pores/channels existing in its contour to interconnect with the surrounding healthy tissue. This hypothesis can corroborate that the angiogenesis of a malignant tumor is an emergency and regulated by the structural and conformational dynamic transformations that occur during TGK, as reported in [1]. On the contrary, if these pores/channels do not exist, the tumor would behave as an isolated system and would self-destruct, in contrast to the experiment.

Fig 3 deserves a careful interpretation, taking into account experimental results reported in the preclinical [1, 3, 9, 11, 14] and clinical [10] studies. The result of Fig 3*A* corresponds with the selection of different values of n_0_/V_0_ in the same TGK for different instants t_0_. For this case, in the experiment is guaranteed fixed c_o_, cell viability, the tumor histological variety and the type of syngeneic host to it. The higher value of n_0_/V_0_ in the same TGK means a larger tumor size, which is reached at a higher t_0_.

Results of Fig 3*B* and 3*C* are associated to the same tumor histological variety that grows in several types of syngeneic hosts to it. For this case, c_o_ and cell viability fixed are guaranteed, taking into account the role of the immune system in the delay of TGK, depending on its immunocompetence degree [10, 11, 22, 35]. As a result, tumors reach different values of n_00_/V_00_ o n_000_/V_000_ at the same time t_0_. The higher value of n_00_/V_00_ (n_0_/V_0_ in Fig 3*B*) or n_000_/V_000_ (n_0_/V_0_ in Fig 3*C*) corresponds to the lower immunocompetence degree of the host (e.g., an immunosuppressed host).

Results of Fig 3*B* refer to two possible situations: 1) different tumor histological varieties that grow in the same type of syngeneic host to them. For this case, c_o_ is different so that each tumor histological variety reaches the same value of n_000_/V_000_ at the same time t_0_. 2) A given tumor histological variety that grows in different types of syngeneic hosts to it. For this case, c_o_ is the same for each tumor histological variety. For these two cases, n_obs_/V_obs_ for each tumor histological variety is reached in a different t_obs_, in accordance with the experiment [9, 11]. These two situations become noticeable when β approaches α (results not shown). Furthermore, this figure reveals that for the highest value of n_obs_/V_obs_ (reached in a greater t_obs_) TGK is slower for n(t) < n_000_ (V(t) < V_000_) and then faster for n(t) > n_000_ (V(t) > V_000_). By contrast, the tumor that has the lowest n_obs_/V_obs_ is the fastest growing for n(t) < n_000_ (V(t) < V_000_) and then its TGK is slowest for n(t) > n_000_ (V(t) > V_000_).

The advantages of GE_8_ over the various formulations of GE [2, 3], the Hahnfeldt model [36–38] and mKJMA equation [1], used to describe undisturbed TGK, are: 1) inclusion of two parameters (n_obs_/V_obs_ y n_000_/V_000_) that are measured and estimated from experimental data. 2) TGK and n_∞_/V_∞_ can be known *a priori* if n_obs_/V_obs_ (starting point of TGK), reached at t_obs_, is estimated for each type of tumor that grows in a syngeneic host to it, as reported in [1, 3, 11].

The relation of the tumor growth with d_f_ and D_f_ is previously obtained by using a mesoscopic formalism and fractal dimension [39]. Besides, Izquierdo-Kurlich [39] report the differences between d_f_ and D_f_ and propose a relation between d_f_ and the dynamic quotient on the interface, named k_c_, (see Eq (48)). This relationship differs from that reported in [4] (see Eq (3)), which is used to obtain Eq (8). If the relation published in [39] is taken into account in this study, Eq (8) is also obtained, except a small change in α numerator (1/2 instead of 1). As a result, 0.75 and 1 are the discontinuities of α, instead of 1 and 1.5, respectively. Nevertheless, these change do not affect significantly the results of this manuscript and confirm that tumors exits for 0 < d_f_ < 1. It can be verified that d_f_ for Ehrlich and fibrosarcoma Sa-37 tumors are less than 0.75 and 1 when Eq (48) in [39] and Eq (3) in [4] are used.

In this study, the tumor growth in the time results of the complex interactions that happen in the tumor and between it and the surrounding healthy tissue, as in [3,14]. Nevertheless, in it does not explicitly discuss the interactions among the individuals neither the cooperative capacity of they in a population to explain its growth behavior, as in [25, 5–8]. These works confirm the fractal property of the tumors, as in this study. Therefore, an additional study may include these interactions for Eq (8).

Further studies can be carried out to validate GE_8_ in TGK of different tumor histological varieties that grow in both immune-competent and immune-deficient organisms. This will allow us to know how D_f_, d_f_, u_2_, V_obs(α,β)_ and V_obs(u2,df,Df)_ change when using different types of tumors and degrees of immune-competence of several organisms, as well as confirming the relationship of these five parameters with the aggressiveness [1], angiogenesis [17], coherence [15, 16], anisotropy, heterogeneity, hardness, changes in the mechanical-elastic-electrical properties of a tumor, among others findings [1].

## Conclusions

GE_8_ describes well the growth kinetics of the Ehrlich and fibrosarcoma Sa-37 tumors and includes two parameters that are directly estimated from the experiment that confirm the fractal property of the tumors and the fractal origin of different Gompertz formulations.

## Appendix A

In [4] it is assumed that the growth ratio of the number *n*(*t*) of tumor cells obeys to the differential equation

$$\frac{dn}{dt}=u_{1}m-u_{2}n,\;n(0)=n_{0},$$ (A1)

where *m* represents the number of tumor cells at the boundary of the tumor, *u*_1_ is the constant of the velocity of the mitosis and *u*_2_ is the constant of the velocity of apoptosis.

Assuming that the boundary has a fractal structure with dimension *d*_*f*_, then $m=k_{1}r^{d_{f}}$, *r* being the average radius of the tumor. On the other side, *n* depends on the morphology of the tumor, described by the fractal dimension *D*_*f*_, and $n=k_{2}r^{D_{f}}$. The morphological constants *k*_1_ and *k*_2_ are related to the magnification of the image [4].

Substituting these values of *m* and *n* and eliminating *r*, Eq (1) can be written as a Bertalanffy-Richards equation.

$$\frac{dn}{dt}=U_{1}\;n^{\theta }-u_{2}\;n=n\;u_{2}\left({\left(\frac{n_{ss}}{n}\right)}^{1-\theta }-1\right),$$

where *n*_*ss*_ = (*u*_2_/*U*_1_)^1/(1−*θ*)^ is the value of *n* at the steady state, the dimensionless morphological parameter θ is defined by *θ* = *d*_*f*_/*D*_*f*_ and U_1_ is given by $U_{1}=u_{1}k_{1}/k_{2}^{\theta }$.

Taking into account that

$$\ln\;x=\lim_{s\to \infty }s(x^{\frac{1}{s}}-1),$$

the above equation is approximated in [4] by the Gompertz equation

$$\left\{ \begin{array}{l} \frac{d\ln(n)}{dt}=u_{2}(\theta -1)\ln\left(\frac{n}{n_{ss}}\right)\\ \ln{\left(n\right)}_{t=0}=0\;n(t=0)=1 \end{array}\right.,$$ (A2)

This approximation is valid when *θ*→1 or *n*→*n*_*ss*_.

In [36] it is justified that the quotient *U*_1_/*u*_2_ can be expressed as a function of *d*_*f*_ and in [4] it is shown that the solution of the differential system (2)

$$n(t)=e^{\frac{\ln\left(U_{1}/u_{2}\right)(1-e^{u_{2}(\theta -1)t})}{1-\theta }}$$

can be expressed as a Gompertz equation (Eq (1) in this paper)

$$n(t)=e^{\left(\frac{\alpha }{\beta }\right)\left(1-e^{-\beta t}\right)}$$

with the intrinsic growth rate of the undisturbed tumor, named α (α > 0), and the deceleration factor, named β (β > 0), related to the tumor fractal dimensions by

$$\left\{ \begin{array}{l} \alpha =u_{2}\left[\ln\frac{U_{1}}{u_{2}}\right]=u_{2}\ln\left(\frac{1.5d_{f}-1}{d_{f}-1}\right)\\ \beta =u_{2}(1-\theta )=u_{2}\left(1-\frac{d_{f}}{D_{f}}\right) \end{array}\right..$$ (A3)

## Supporting information

S1 Data Supporting information. (TXT)
